# Harnessing Corneal Stromal Regeneration for Vision Restoration: A Comprehensive Review of the Emerging Treatment Techniques for Keratoconus

**DOI:** 10.7759/cureus.69835

**Published:** 2024-09-21

**Authors:** Zoya Javed, Sachin Daigavane

**Affiliations:** 1 Ophthalmology, Jawaharlal Nehru Medical College, Datta Meghe Institute of Higher Education and Research, Wardha, IND

**Keywords:** cell-based therapies, corneal cross-linking, corneal stromal regeneration, gene therapy, keratoconus, tissue engineering

## Abstract

Keratoconus is a progressive corneal disorder characterized by thinning and conical protrusion, leading to visual impairment that often necessitates advanced treatment strategies. Traditional management options, including corrective lenses, corneal cross-linking (CXL), and surgical interventions such as corneal transplants and intracorneal ring segments (ICRS), address symptoms but have limitations, especially in progressive or advanced cases. Recent advancements in corneal stromal regeneration offer promising alternatives for enhancing vision restoration and halting disease progression. This review explores emerging techniques focused on corneal stromal regeneration, emphasizing cell-based therapies, tissue engineering, and gene therapy. Cell-based approaches, including corneal stromal stem cells and adipose-derived stem cells, are promising to promote tissue repair and functional recovery. Tissue engineering techniques, such as developing synthetic and biological scaffolds and 3D bioprinting, are being investigated for their ability to create viable corneal grafts and implants. Additionally, gene therapy and molecular strategies, including gene editing technologies and the application of growth factors, are advancing the potential for targeted treatment and regenerative medicine. Despite these advancements, challenges remain, including technical limitations, safety concerns, and ethical considerations. This review aims to provide a comprehensive overview of these innovative approaches, highlighting their current status, clinical outcomes, and future directions in keratoconus management.

## Introduction and background

Keratoconus is a progressive ocular disorder characterized by the thinning and conical protrusion of the cornea [1]. This deformation leads to significant visual impairment due to irregular astigmatism and myopia. The prevalence of keratoconus varies globally, with estimates ranging from one in 2,000 to one in 10,000 individuals [2]. The condition typically manifests in the late teens to early twenties and is more common in individuals of Middle Eastern, South Asian, and Jewish descent. Although keratoconus often affects both eyes, it can present with asymmetry in severity, causing a range of visual disturbances [3]. The pathophysiology of keratoconus involves a complex interplay of genetic, environmental, and biochemical factors that contribute to the degradation of corneal stromal tissue. This degradation results in the loss of corneal collagen and alterations in the extracellular matrix, weakening the corneal structure and leading to its progressive thinning and bulging. The disease progression is generally categorized into early, moderate, and advanced stages, with varying degrees of visual distortion and functional impairment [4]. The rate at which keratoconus progresses can differ significantly between individuals, with some experiencing rapid deterioration while others maintain relatively stable vision for longer periods [5].

Current treatment approaches for keratoconus vary depending on the stage and severity of the condition. In the early stages, corrective lenses often manage visual symptoms [6]. Glasses may be effective for mild cases, but as the condition advances, specialized contact lenses such as rigid gas permeable (RGP) or scleral lenses are usually required to address the irregular corneal surface and improve visual acuity. These lenses help create a regular optical surface and enhance comfort [6]. Corneal cross-linking (CXL) is a minimally invasive procedure designed to halt the progression of keratoconus. It involves applying riboflavin (vitamin B2) to the cornea and then exposing it to ultraviolet (UV) light [7]. This process induces the formation of new cross-links between collagen fibers in the corneal stroma, which increases the cornea's biomechanical stability and prevents further thinning. CXL has become a cornerstone in managing progressive keratoconus, particularly in patients without advanced disease [8].

Surgical interventions may be necessary for advanced cases of keratoconus where non-surgical treatments are no longer effective. These include corneal transplants, such as penetrating or lamellar keratoplasty, which involve replacing damaged corneal tissue with a donor cornea [9]. While these procedures can restore visual function, they carry risks of graft rejection and other complications. Intracorneal ring segments (ICRS) are another surgical option involving the insertion of ring-shaped implants into the corneal stroma to flatten the cornea and improve visual acuity. Although ICRS do not stop disease progression, they can provide temporary visual improvement and reduce reliance on contact lenses [10]. This review explores emerging techniques for treating keratoconus, specifically corneal stromal regeneration. As traditional treatment options have limitations, new approaches leveraging advances in cell-based therapies, tissue engineering, and gene therapy hold promise for restoring corneal function and improving patient outcomes. By examining these innovative strategies, this review provides a comprehensive overview of current advancements and future directions in keratoconus management.

## Review

Corneal stromal regeneration

The corneal stroma, also known as the substantia propria, is the thickest layer of the cornea, accounting for approximately 90% of its total thickness [11]. This layer comprises a highly organized arrangement of collagen fibrils and proteoglycans, structured around 200 flattened lamellae [12]. The precise organization of these components is vital for maintaining corneal transparency and mechanical strength. The primary collagen types found in the stroma are types I and V, which are aligned to minimize light scattering, thus ensuring optimal vision [13]. In addition to maintaining transparency, the stroma is crucial for ocular immunity and provides essential structural support to the cornea, which is critical for its refractive function. The unique composition and organization of the extracellular matrix (ECM) within the stroma are key to its transparency, shape, and resistance to deformation under pressure [13]. In keratoconus cases, the corneal stroma's normal architecture is disrupted, leading to disorganized lamellae and progressive corneal thinning. This condition causes a conical deformation that significantly impairs visual acuity [1]. The disorder is marked by a loss of structural integrity due to altered collagen organization and proteoglycan composition, which weakens the biomechanical properties of the cornea. As the disease advances, patients may experience severe visual distortion and increased sensitivity to light, requiring prompt intervention [1]. Stromal regeneration involves intricate cellular and molecular processes essential for restoring corneal integrity. Keratocytes, the primary cells within the stroma, play a critical role in maintaining and remodeling the ECM. Following injury or in conditions like keratoconus, keratocytes can transform into myofibroblasts. While myofibroblasts are crucial for wound healing, their uncontrolled activity can lead to fibrosis and further compromise corneal structure [14]. The regeneration process is regulated by various growth factors, cytokines, and extracellular matrix components that facilitate the recruitment and activation of keratocytes. Key factors in this process include transforming growth factor-beta (TGF-β), which regulates collagen synthesis and ECM remodeling, and various proteoglycans that influence cell behavior and ECM assembly [15]. Several factors significantly influence the efficiency of stromal regeneration. The composition of the extracellular matrix is paramount, as the balance of collagen types and proteoglycans is crucial for effective healing [16]. Alterations in the expression of small leucine-rich proteoglycans (SLRPs) can affect collagen fibrillogenesis, thereby impacting the structural integrity of the stroma. Additionally, the cellular environment plays a vital role; inflammatory cytokines and growth factors can promote or inhibit keratocyte activity and ECM remodeling [17]. For example, excessive signaling from TGF-β can lead to pathological scarring instead of normal regeneration. Finally, mechanical forces, such as intraocular pressure and external stresses, can influence the stroma's ability to regenerate and maintain its structure after injury. Understanding these mechanisms is critical for developing effective treatments for keratoconus, particularly those aimed at promoting corneal stromal regeneration and restoring visual function [17].

Emerging techniques

Cell-Based Therapies

Cell-based therapies are emerging as a promising approach for treating keratoconus by focusing on regenerating the corneal stroma and restoring its structural integrity. Two significant types of stem cells currently under investigation are corneal stromal stem cells and adipose-derived stem cells (ADSCs) [18]. Corneal stromal stem cells, a rare population in the peripheral cornea and limbus, have gained attention for their potential in regenerative therapies. These cells can be isolated using specific surface markers derived from neural crest cells, allowing them to self-renew and differentiate into keratocytes, essential for maintaining corneal structure and function [19]. Research suggests that transplanting these stem cells could replenish the keratocyte population in corneas affected by keratoconus. Preclinical studies have demonstrated that when injected into the corneal stroma, these cells can differentiate into adult keratocytes and produce collagen. Animal models, such as mice, have shown that these stem cells do not provoke immune rejection, indicating their potential for safe application in humans. However, further research is needed to establish standardized protocols for their clinical use, including identifying optimal cell sources, refining delivery methods, and determining patient selection criteria [20]. Adipose-derived stem cells (ADSCs) represent another promising avenue for keratoconus treatment. Harvested from adipose tissue, these stem cells are known for their regenerative properties. ADSCs can differentiate into various cell types, including keratocytes, and secrete growth factors that support tissue repair and regeneration. The therapeutic effects of ADSCs are mediated through the secretion of paracrine factors that enhance the survival and function of resident corneal cells and through their direct differentiation into keratocytes [21]. Clinical trials have demonstrated the efficacy of ADSCs in treating advanced keratoconus. In one study, autologous ADSCs were injected into corneal pockets created by femtosecond laser in patients with advanced keratoconus. The results showed that the injected stem cells remained viable for at least six months and successfully synthesized new collagen, suggesting their potential to restore corneal structure. These findings indicate that ADSCs could be a viable treatment option, especially in cases where traditional surgical interventions are either unsuitable or unavailable [22].

Tissue Engineering Approaches

Tissue engineering revolutionizes regenerative medicine by developing synthetic and biological scaffolds and advanced bioengineering strategies. Synthetic scaffolds are often constructed from materials like polylactic acid (PLA), polyglycolic acid (PGA), and poly(lactic-co-glycolic acid) (PLGA) [23]. These materials are preferred for their controlled degradation rates, favorable mechanical properties, and the ability to be customized for specific applications. They can be precisely engineered to provide optimal support for cell growth and tissue regeneration [23]. In contrast, biological scaffolds are derived from natural extracellular matrix (ECM) components such as collagen, laminin, and chitosan. These scaffolds are renowned for their superior biocompatibility and bioactivity, which promote cell attachment and proliferation. Typically, they are created through decellularization processes that remove cellular components while preserving the ECM's structural integrity [24]. Ongoing research is investigating the effectiveness of both synthetic and biological scaffolds across various tissue engineering applications, including bone and cartilage regeneration, wound healing, and organ repair. Studies underscore the critical role of scaffold design, particularly pore size and architecture, significantly impacting cell behavior and tissue integration [25]. 3D bioprinting represents an innovative bioengineering strategy that enables the precise fabrication of scaffolds with complex geometries that closely mimic natural tissues. This technique allows for the incorporation of living cells and biomaterials in a layer-by-layer fashion, facilitating the creation of customized scaffolds tailored to the specific needs of patients [26]. Current research is focused on optimizing bioink formulations and refining printing techniques to enhance cell viability and functionality within the printed constructs. Additionally, bioengineering strategies include the development of customized grafts and implants that can be tailored to individual anatomical and physiological requirements. These grafts are often produced using patient-specific imaging data to ensure an optimal fit, thereby improving integration and reducing the risk of complications. Researchers are also exploring hybrid materials that combine synthetic and natural components to leverage the benefits of both scaffold types, enhancing mechanical properties while promoting biological functionality [27].

Gene Therapy and Molecular Techniques

**Figure 1:**
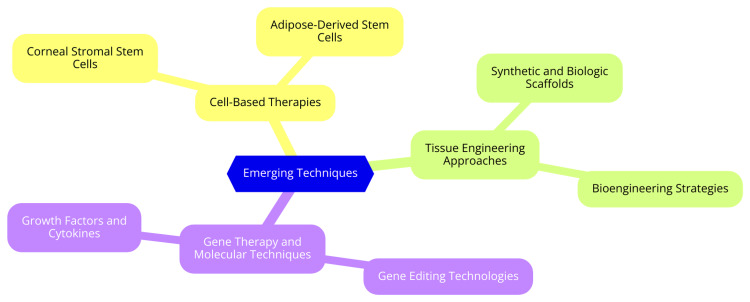
Emerging techniques Image Credit: Dr. Zoya Javed

Gene editing technologies, particularly CRISPR/Cas9, have revolutionized genetics and offer significant promise for treating ocular diseases, including keratoconus. CRISPR/Cas9 allows for precise genome modifications, enabling researchers to target specific genes related to corneal structure and function. This technology can potentially correct genetic mutations contributing to keratoconus, offering a novel therapeutic approach [28]. Other gene editing methods, such as transcription activator-like effector nucleases (TALENs) and zinc finger nucleases (ZFNs), also provide possibilities for targeted gene therapy. These methods could be applied in keratoconus to repair or modify genes associated with corneal thinning and irregular shape, thereby restoring normal corneal architecture and function [29]. The application of gene editing in keratoconus could focus on several key areas: correcting genetic mutations that predispose individuals to keratoconus, thereby preventing or slowing disease progression; enhancing corneal regeneration by upregulating growth factors or proteins that promote healing; and enabling personalized medicine by tailoring treatments to an individual's genetic profile to optimize therapeutic efficacy [30]. Growth factors play a critical role in corneal repair and regeneration. Important growth factors involved in corneal epithelial healing include epidermal growth factor (EGF), which enhances epithelial cell proliferation and migration; hepatocyte growth factor (HGF), which promotes cell proliferation and has anti-inflammatory properties; vascular endothelial growth factor (VEGF), which is involved in angiogenesis and supports corneal nerve regeneration; and transforming growth factor-beta (TGF-β), which regulates various cellular processes, including inflammation and fibrosis, and is essential in the wound healing response [31]. The administration of these growth factors has shown significant effects on corneal regeneration. They can accelerate wound healing by promoting cell proliferation and migration, enhance structural integrity through remodeling the extracellular matrix, and regulate inflammation by modulating inflammatory responses [32]. However, challenges remain in fully understanding their signaling pathways and optimizing their therapeutic applications. Continued research is essential to explore the potential of these molecular techniques in effectively managing keratoconus and to develop targeted therapies that can improve patient outcomes [32]. Emerging techniques are illustrated in Figure 1.

Clinical outcomes and challenges

Recent clinical trials have highlighted the efficacy and safety of various treatments for keratoconus, with a particular focus on corneal collagen cross-linking (CXL) [33]. For example, the EpiSmart crosslinking study reported significant improvements in corrected distance visual acuity (CDVA) and uncorrected distance visual acuity (UCVA) at both six and 12 months post-treatment, demonstrating a strong safety and efficacy profile [34]. Long-term data from the Save Sight Keratoconus Registry (SSKR) also support these findings, indicating a high likelihood of keratoconus stability or improvement five years after CXL treatment. This study showed that both standard (sCXL) and accelerated (aCXL) protocols were effective, with sCXL offering better long-term outcomes compared to aCXL [35]. Extended follow-ups have confirmed that CXL patients experienced improved visual acuity and corneal topography. Many patients maintained stable or improved keratoconus conditions, reinforcing the long-term effectiveness of this treatment. This is crucial as it reassures patients and clinicians regarding the durability of CXL outcomes [35]. However, despite these advancements, several challenges persist in treating keratoconus. Technical barriers include variability in treatment protocols and individual patient responses to CXL, which can result in inconsistent outcomes [36]. Factors such as individual healing responses and corneal biomechanics further complicate treatment efficacy. Consequently, there is an increasing need for personalized treatment approaches. Researchers are exploring theranostic-guided CXL, which aims to customize treatments based on individual patient profiles, thereby enhancing overall outcomes [36]. Ethical and regulatory challenges also play a critical role in advancing keratoconus treatments. Clinical trials must adhere to strict ethical guidelines, ensuring informed consent and patient safety. The regulatory processes required for approving new treatment modalities can be lengthy, potentially delaying access to innovative therapies [37]. Additionally, the ethical implications of emerging technologies, such as gene therapy and tissue engineering, require careful consideration to balance potential benefits against risks. Addressing these ethical and regulatory challenges is essential for fostering trust and ensuring the responsible development of new treatments [38]. Clinical outcomes and challenges in keratoconus treatment with corneal stromal regeneration techniques are detailed in Table 1.

**Table 1:** Clinical outcomes and challenges in keratoconus treatment with corneal stromal regeneration techniques

Aspect	Clinical Outcome	Challenges
Stem Cell Therapy [39]	Improved corneal clarity and vision recovery in early-stage keratoconus	Limited availability of stem cells, ethical concerns, and potential for immune rejection
Corneal Cross-linking (CXL) [40]	Stabilizes corneal structure and halts the progression of keratoconus	Requires precision in the application; long-term effects still under investigation
Tissue Engineering [41]	Bioengineered corneal tissues show promise in early clinical trials for stromal regeneration	High cost, limited scalability, and regulatory hurdles for widespread clinical use
Nanotechnology for Drug Delivery [42]	Enhanced precision in delivering therapeutic agents directly to the cornea, improving treatment outcomes	Potential toxicity and unknown long-term effects of nanoparticles on ocular tissues
Gene Therapy [43]	Potential to correct genetic mutations causing keratoconus, offering a long-term solution	Complexity of gene editing techniques, risk of off-target effects, and ethical concerns
Artificial Corneal Implants [44]	Successful restoration of vision in patients unsuitable for corneal transplants	Biocompatibility issues, high rejection rates, and need for further research on long-term outcomes
3D Bioprinted Corneas [45]	Personalized grafts show potential for a perfect anatomical fit, improving post-surgical outcomes	Technological limitations in bioprinting, high costs, and lack of clinical standardization
Photochemical Corneal Strengthening [40]	Non-invasive procedures result in minimal side effects and effective corneal stabilization	Limited by the availability of technology and the need for further refinement for broader patient applicability
Combination Therapies [46]	Combining cross-linking with stem cell or gene therapies offers synergistic benefits for halting progression	Coordinating multiple therapies increases complexity, costs, and potential for adverse effects
Immunomodulation [47]	Reduced risk of rejection in corneal grafts, leading to better graft survival rates	Developing safe, long-term immunosuppressive strategies without increasing infection risks is a challenge

Future directions

One of the most promising research areas in keratoconus treatment involves advancements in biomaterials and cell therapies. Developing innovative biomaterials is essential for creating scaffolds that support corneal regeneration. Researchers are investigating hydrogels and other biocompatible materials designed to mimic the natural extracellular matrix of the cornea [48]. These materials promote cell adhesion, proliferation, and differentiation, facilitating effective corneal tissue healing and regeneration. Additionally, stem cell therapy is emerging as a potential treatment for keratoconus. Research into using limbal stem cells and mesenchymal stem cells aims to restore corneal integrity and function, offering a regenerative approach that could reduce corneal transplants and improve long-term outcomes [49]. Another exciting area of innovation is integrating artificial intelligence (AI) and machine learning in treatment planning. AI and machine learning algorithms can analyze extensive datasets to predict disease progression in keratoconus patients. By identifying patterns and risk factors, these technologies assist clinicians in making informed decisions regarding treatment options and timing [50]. Moreover, AI can personalize treatment plans by considering various factors, including corneal topography, genetic predispositions, and lifestyle. This personalized approach enhances treatment efficacy and minimizes complications, improving patient outcomes [51]. Advancements in keratoconus treatment are poised to significantly enhance patient care, particularly in outcomes and quality of life. With the introduction of advanced treatment modalities, patients will likely experience substantial improvements in visual acuity and overall eye health [52]. Techniques that promote corneal regeneration and stability can lead to better functional vision and reduce reliance on corrective lenses. Furthermore, innovative treatments, especially less invasive ones, can lower the risk of complications associated with traditional surgeries. Reducing adverse events contributes to a smoother recovery and greater patient satisfaction [52]. Personalized treatment approaches will also be crucial in advancing patient care. Integrating AI and biomaterials allows a more nuanced understanding of each patient’s condition. Tailored treatment plans, developed based on individual corneal characteristics, lifestyle factors, and preferences, ensure that interventions are more effective and aligned with patient needs [53]. This personalized approach can enhance patient engagement and compliance, as individuals are more likely to adhere to prescribed therapies and follow-up care when they understand that their treatment is specifically designed for them [53]. Future directions in keratoconus treatment with a focus on corneal stromal regeneration are detailed in Table 2.

**Table 2:** Future directions in keratoconus treatment with a focus on corneal stromal regeneration

Aspect	Future Direction	Potential Impact
Stem Cell Therapies [54]	Exploration of mesenchymal stem cells for regenerating corneal stroma	Promotes tissue repair and regeneration, reducing the need for corneal transplants
Advanced Cross-linking Techniques [55]	Development of enhanced cross-linking protocols with minimal side effects	Improved corneal stability with fewer complications and better long-term results
Tissue Engineering [56]	Bioengineered corneal implants utilizing biomaterials for stromal reconstruction	Potential to restore corneal function without the need for donor tissue
Nanotechnology Applications [57]	Use of nanoparticles to deliver growth factors and regenerative molecules to the cornea	Targeted regeneration with enhanced precision, minimizing damage to surrounding tissue
Gene Therapy [58]	Investigation of gene-editing techniques to address genetic predisposition to keratoconus	Prevents the progression of keratoconus at the molecular level, offering a personalized treatment approach
Artificial Corneas [59]	Development of synthetic corneal substitutes that mimic natural corneal properties	Provides an alternative for patients unsuitable for corneal transplants, reducing global dependency on donors
3D Bioprinting [60]	Application of 3D bioprinting for creating personalized corneal scaffolds	Customized corneal tissue generation that matches individual patients’ needs, improving surgical outcomes
Photochemical Treatments [40]	Exploring non-invasive photochemical methods for corneal strengthening without the need for UV light	Reduces potential damage from UV light exposure, making treatments safer and more accessible
Immunomodulatory Therapies [61]	Development of therapies that minimize immune rejection of corneal grafts and implants	Enhances graft survival rates and reduces complications from immune system reactions
Personalized Medicine Approaches [62]	Utilization of genetic and biomarker profiling to tailor treatments to individual patients	Improves treatment efficacy by customizing therapeutic strategies based on individual genetic makeup and disease progression

## Conclusions

In conclusion, the management of keratoconus has evolved significantly with the introduction of advanced techniques aimed at halting disease progression and restoring corneal function. Traditional treatments, such as corrective lenses and corneal cross-linking, have managed the condition and improved patient outcomes. However, emerging techniques focusing on corneal stromal regeneration offer promising avenues for more effective treatment. Innovations in cell-based therapies, tissue engineering, and gene therapy are paving the way for novel approaches that address the underlying pathophysiology of keratoconus, potentially transforming the standard of care. As research progresses, these advancements hold the potential to enhance visual outcomes, improve quality of life for patients, and ultimately offer more personalized and effective treatment options. Continued exploration and clinical validation of these emerging techniques will be crucial in shaping the future of keratoconus management and providing hope for those affected by this challenging condition.
